# Myoepithelial Cell-Rich Pleormorphic Adenoma of Minor Salivary Gland of Parapharyngeal Space

**DOI:** 10.1155/2012/537381

**Published:** 2012-08-22

**Authors:** Digvijay Singh Rawat, Divij Sonkhya, Nishi Sonkhya, Shubha Gupta

**Affiliations:** ^1^Department of E.N.T, S.M.S. Medical College and Hospital, Jaipur, Rajasthan 302004, India; ^2^Department of E.N.T, N.K.P. Salve Institute and Lata Mangeshkar Hospital, Nagpur, Maharashtra 440019, India; ^3^S.D.M. Hospital, Jaipur, Rajasthan 302004, India

## Abstract

Parapharyngeal space tumors are rare and constitute only 0.5–1.0% of head and neck tumors. Minor salivary gland tumor is still rare in parapharyngeal space. We are reporting a case of pleomorphic adenoma of minor salivary gland of parapharyngeal space. A 42-year-old female presented with a history of mass in the oropharynx for 3 years. She presented with “hot potato voice” and dysphagia. CECT and MRI were done, showing large parapharyngeal space tumor. FNAC was suspicious for tumor of nerve cell origin. Tumor was excised using “paramedian mandibulotomy with mandibular swing approach”. Histopathological examination was inconclusive, suggesting possibility of extraskeletal myxoid chondrosarcoma, solitary fibrous tumor, neurogenic tumor. On immunohistochemistry, tumor was positive for cytokeratin, EMA (dim), S-100, and P 63 and negative for SMA thus proving the case as myoepithelial cell-rich pleomorphic adenoma.

## 1. Introduction 

Parapharyngeal space tumors are rare [1, 2]. Pleomorphic adenomas are the most common parapharyngeal tumors and present as slowly increasing painless mass in neck and/or retrotonsillar area causing dysphagia, hoarseness, otalgia, and difficulty in breathing [1, 3]. Pleomorphic adenomas in the parapharyngeal space usually arise from deep lobe of the parotid, but rarely can develop de novo from displaced or aberrant salivary gland tissue within a lymph node [2]. We are reporting a case of large parapharyngeal space tumor, presented with dysphagia, “hot potato voice,” and difficulty in breathing. The tumor was initially suspected to be of neurogenic origin on FNAC. Histopathology and immunohistochemistry proved it to be a rare tumor of minor salivary gland origin, myoepithelial cell-rich pleomorphic adenoma.

## 2. Case Report

**Figure 1:**
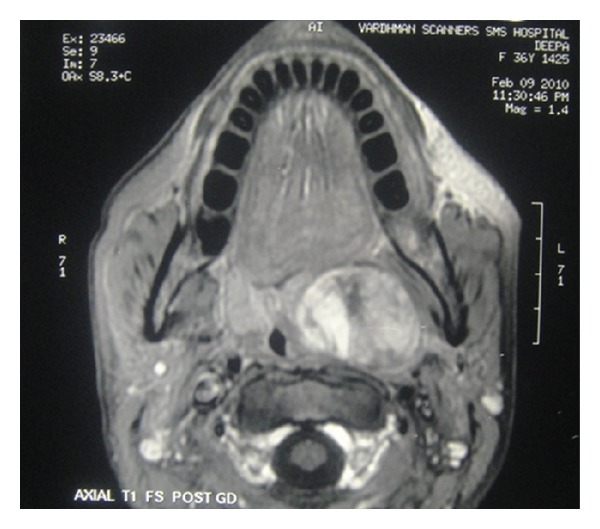
T1-weighted post-Gd MRI large showing heterogenous mass in left parapharyngeal space.

A 42 years old female presented with painless slowly progressing mass in the oropharynx for 3 years. There was a history of difficulty in deglutition for 1 year and difficulty in breathing for 3 months. Patient was nonsmoker and nonalcoholic. On clinical examination a large mass occupying the whole of the oropharynx arising from the left lateral pharyngeal wall crossing the midline and almost touching the opposite side was observed. Neurological examination was normal. Patient consulted for swelling in oropharynx 2 years ago, she was investigated and advised for surgery but she was lost to followup. MRI with Gadolinium enhancement (dated February 2009-2010) showed well-defined heterogenous mass occupying left parapharyngeal space (Figure 1). 

**Figure 2:**
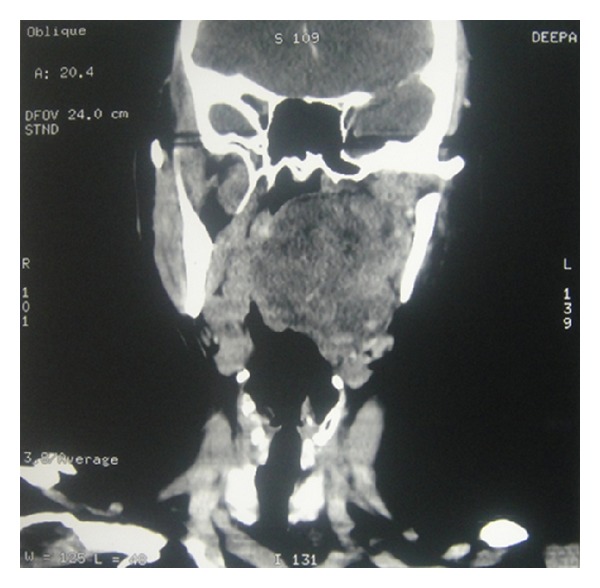
CECT coronal cut showing extent of mass.

**Figure 3:**
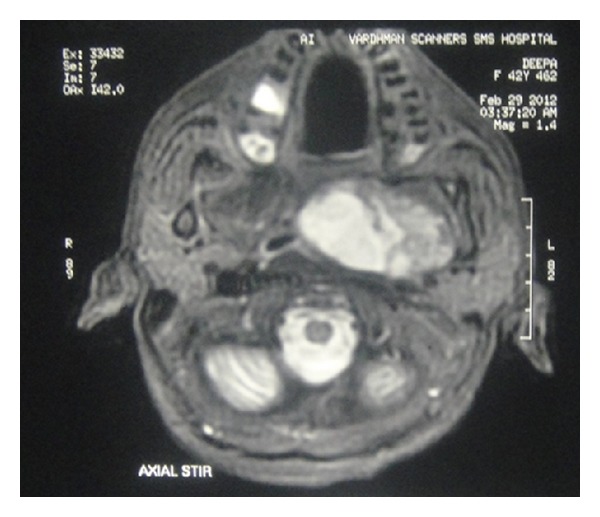
T1-weighted post-Gd MRI image showing preservation of a fat plane between the tumor and parotid gland.

**Figure 4:**
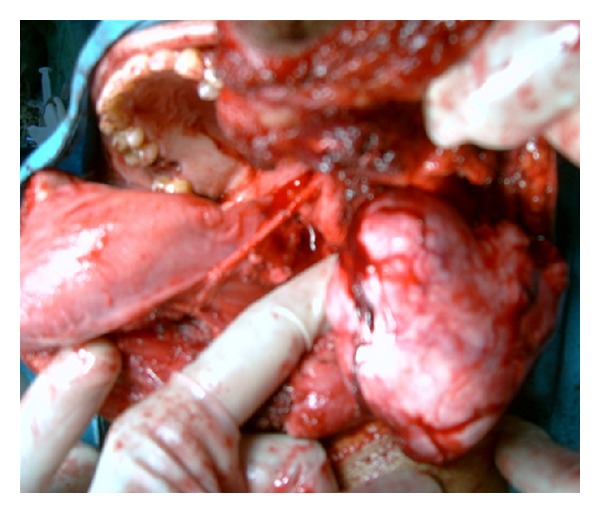
Tumor being delivered by “paramedian mandibulotomy with mandibular swing” approach.

**Figure 5:**
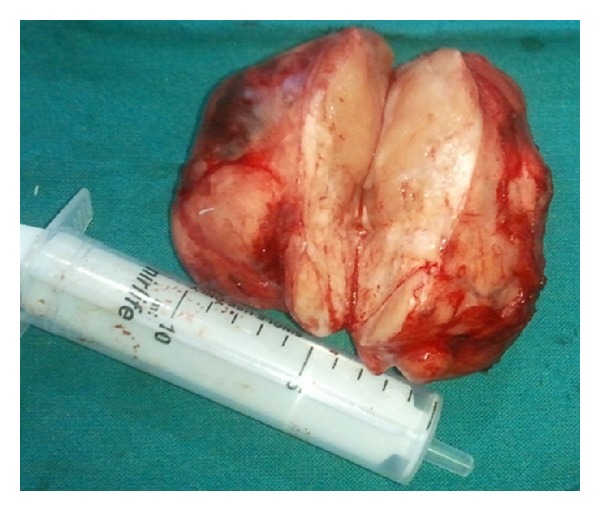
Excised tumor specimen with cut section.

Fresh radiological imaging was performed. CECT showed a large heterogenous mass in the left parapharyngeal region extending from level of nasopharynx to the level of C3 vertebra inferiorly (Figure 2). MRI with Gd showed large heterogeneously enhancing mass measuring 72 × 44 × 66 mm in left parapharyngeal space causing marked mass effect over nasooropharyngeal air column from left side with effacement. T1-weighted image showed preservation of a fat plane between the tumor and the parotid (Figure 3). Excision of the tumor was done by “paramedian mandibulotomy with mandibular swing” approach (Figure 4). Gross examination revealed a firm nodular mass of 8 × 7 × 5 cm, cut surface was solid greyish white (Figure 5). Postoperative period was uneventful.

Histopathological examination showed a mesenchymal neoplasm with a myxoid background. Cells were small and oval and arranged in streaks with minimal mitotic activity and anaplasia. Impression was of mesenchymal tumor of benign/low-grade malignant potential. The differential diagnosis suggested the possibility of extraskeletal myxoid chondrosarcoma solitary fibrous tumor neurogenic tumor. On immunohistochemistry, tumor was positive for cytokeratin, EMA (dim), S-100, and P63 and negative for SMA thus proving the case as myoepithelial cell-rich pleomorphic adenoma.

## 3. Discussion

Pleomorphic adenoma is a benign salivary gland tumor, also known as “benign mixed tumor” having epithelial, myoepithelial, and stromal components. Pleomorphic adenoma is the most common salivary gland tumor in both children and adults [4]. Pleomorphic adenoma is seen more often in females than in males (2 : 1 ratio) [5]. 5–10% of cases of pleomorphic adenomas involve minor salivary glands. The most common site of pleomorphic adenoma of the minor salivary glands is the palate followed by lip, buccal mucosa, floor of mouth, tongue, tonsil, pharynx, retromolar area, and nasal cavity [2]. Simultaneous pleomorphic adenomas in the parotid gland and minor salivary gland at the parapharyngeal space has been reported [6].

MRI is the method of choice for imaging the parapharyngeal space. T1-weighted images are best for demonstrating normal anatomy and any tumor-fat interfaces. Deep lobe parotid tumors differentiated from ectopic salivary gland tumors by the preservation of a fat plane between the tumor and the parotid [7, 8]. This was also observed in our case proving it to be of minor salivary gland origin.

Although histopathology remains the gold standard, owing to the difficult surgical approach of tumors in parapharyngeal space FNAC is usually done [9]. A nondiagnostic aspirate has been reported in 25–60% of cases and is most frequently due to lack of cellular material, excessive bleeding, and other technical problems relating to adequately targeting the lesion [10]. USG/CT-guided FNAC has proved to be useful both in terms of morphological analysis and efficacy [9]. Open transoral biopsies should be avoided in all cases [10].

The majority of parapharyngeal tumors can be excised via the cervical and the cervicoparotid approach. Mandibulotomy improves surgical access to the parapharyngeal space and considered for selected patients to improve visual assessment and vascular control. These cases include malignant neoplasms, selected recurrent neoplasms, very large benign neoplasms, highly vascular neoplasms, and skull base erosions [10]. We performed mandibulotomy as the tumor was very large and going up to skull base.

The key histopathological features of pleomorphic adenoma are a variable pattern of epithelium in a loosely fibrous stroma which may be myxoid, chondroid, or mucoid. In the minor salivary glands, lesions are often more solid or cellular than those seen in the major glands, and the myoepithelial cells are often polygonal with a pale eosinophilic cytoplasm giving an epithelioid or plasmacytoid phenotype [11].

Myoepithelial cells are an important component of salivary gland tumors and are partly responsible for their diverse histology. Detection of myoepithelial differentiation both in aspiration cytology and histologic examination can also help diagnosing a tumor as of salivary gland type [12]. The proteins SMA, calponin, CD29, S100, and P63, which are present from the earliest stage of maturation of myoepithelial cell are the most valuable for their diagnosis [13]. On immunohistochemistry, in our case tumor was positive for CK, EMA, S-100, and p63 and negative for SMA, thus proving the case as pleomorphic adenoma with predominantly myoepithelial cells type.
